# QTc interval prolongation in pediatric eating disorder population

**DOI:** 10.3389/fped.2026.1854024

**Published:** 2026-06-26

**Authors:** Emily Kacer, Erin Burnley, Terrel Marshall, Clarissa Ngo, Hon Yiu So, Allison Rodrigues, Narayanaswamy Balakrishnan, Tapas Mondal

**Affiliations:** 1 Michael G. DeGroote School of Medicine, McMaster University, Hamilton, ON, Canada; 2 Department of Mathematics and Statistics, Oakland University, Rochester, MI, United States; 3 Department of Pediatrics, Division of Adolescent Medicine, Health Sciences Centre, McMaster University, Hamilton, ON, Canada; 4 Department of Mathematics and Statistics, McMaster University, Hamilton, ON, Canada; 5 Department of Mathematics, Atilim University, Ankara, Türkiye; 6 Department of Pediatrics, Division of Cardiology, Health Sciences Centre, McMaster University, Hamilton, ON, Canada.

**Keywords:** eating disorder, electrolytes, medications, pediatric, QTc interval

## Abstract

**Introduction:**

Eating disorders in pediatric populations are associated with several cardiac complications, including QTc prolongation, which can lead to fatal arrhythmias. While some studies suggest eating disorders inherently lead to QTc prolongation, emerging evidence suggests extrinsic factors may play a greater role.

**Methods:**

This retrospective chart review evaluates QTc interval prolongation in pediatric patients with eating disorders at a tertiary children's hospital. Demographic factors, ECGs, electrolyte levels, and the use of QTc-prolonging medications were analyzed to evaluate their association with QTc prolongation.

**Results:**

Among 435 patients, 405 had normal QTc intervals (<440 ms), 8 were borderline prolonged (440–460 ms), and 22 had prolonged intervals (>460 ms) using the Bazett calculation. Mean QTc values differed significantly across eating disorder subtypes (*p* = 0.0063), with the longest values observed in bulimia nervosa and binge eating disorder, and the shortest in anorexia nervosa. Patients taking one or more QTc-prolonging medications had a longer mean QTc interval than those not taking medications (*p* < 0.001). There were significant inverse correlations between serum calcium (*p* = 0.0001), magnesium (*p* = 0.01), and potassium (*p* = 0.003) levels and QTc length.

**Discussion:**

These findings suggest QTc-prolonging medications and electrolyte abnormalities can be associated with longer QTc intervals in pediatric patients with eating disorders and should continue to be monitored as modifiable risk factors.

## 1 Introduction

Eating disorders are serious, physical and mental health illnesses that disproportionately impact children and adolescents (1). The increasing prevalence of pediatric eating disorder diagnoses and hospitalizations constitutes a critical public health concern, as these conditions can profoundly impact a youth's development and be associated with considerable chronicity, morbidity, and mortality (2, 3).

The most common eating disorders seen in youth are anorexia nervosa (AN), bulimia nervosa (BN), avoidant/restrictive food intake disorder (ARFID), other specified feeding or eating disorder (OSFED), and binge-eating disorder (BED). While most research has focused on the health impacts of malnutrition in AN and purging in BN, we now know that OSFED and ARFID have also been connected with multiorgan morbidity (2–4). Of particular concern are the cardiovascular effects associated with eating disorders including bradycardia, hypotension, decreased left ventricular mass, and most controversially, QTc prolongation (5).

AN is an eating disorder characterized by restriction in nutritional intake, intense fear of weight gain, and a distorted body image, leading to low body weight (6). Malnutrition can have profound effects on the cardiovascular system, with complications including bradycardia, hypotension, and arrhythmias, largely due to electrolyte imbalances, loss of cardiac muscle mass, and autonomic dysfunction (2, 7, 8). AN also has the highest mortality rate of all eating disorders (2).

BN is defined by recurrent episodes of binge eating followed by inappropriate compensatory purging behaviors such as vomiting, excessive exercise, or laxative misuse (9). These behaviors can also lead to significant electrolyte imbalances, including hypokalemia, hypophosphatemia, and hypomagnesemia, which can disrupt cardiac repolarization (2, 9).

ARFID is an eating disorder characterized by a persistent failure to meet appropriate nutritional requirements due to a lack of interest in eating, or an avoidance of certain foods based on sensory characteristics, and/or a fear of adverse consequences of eating (3). Although ARFID is not driven by body image concerns, the resulting malnutrition and low body weight can lead to similar medical complications as in AN including electrolyte disturbances (3). Generally, heart rate and blood pressure are normal in these patients (3). Contrasting ARFID, BED is characterized by recurrent episodes of excessive food consumption within a short period, accompanied by a loss of control but without any compensatory behaviors to prevent weight gain (10). BED is often associated with obesity, metabolic syndrome, and increased risk of cardiovascular events (10).

OSFED is a heterogenous diagnosis reserved for individuals with significant disordered eating behaviors who do not meet the full criteria for AN, BN or BED (11). OSFED can result in severe medical complications, particularly when behaviors like purging, restrictive eating, or excessive exercise are present. Finally, rumination disorder involves repeated regurgitation of food, which may be re-chewed, re-swallowed, or spit out and can also potentially result in malnutrition (12).

The exact mechanism by which eating disorders, such as AN and BN, prolong the QTc interval remains unclear (13). It has been postulated that myocardial repolarization may be altered by eating disorder pathophysiology (14–16). Studies show mixed findings regarding QTc prolongation in adolescent eating disorder populations, with some demonstrating no significant increase compared to control groups (14–22). Average QTc values for pediatric eating disorder patients generally do not exceed clinically significant durations for prolongation (14–23). Increasingly, the literature does not support inherent QTc prolongation in eating disorders and instead highlights extrinsic factors associated with disordered eating in both pediatric and adult populations (7, 19, 23, 24).

The QTc interval, which corrects the QT interval on an electrocardiogram (ECG) for heart rate, is an important indicator of cardiac repolarization. In pediatric populations, QTc values under 440 milliseconds are considered normal, values between 440 and 460 milliseconds are categorized as borderline, and values above 460 milliseconds are deemed prolonged (25). Prolonged QTc intervals are clinically relevant as they increase the risk of torsades de pointes (TdP), a life-threatening arrhythmia that may lead to sudden cardiac death (26).

Re-feeding is a critical component in the treatment of eating disorders with nutritional rehabilitation and weight restoration being essential for recovery. However, nutritional rehabilitation can lead to the potentially life-threatening condition known as re-feeding syndrome. This is a syndrome with both clinical and biochemical derangements triggered by a surge in insulin after the reintroduction of a carbohydrate load following a period of starvation (27). This shift drives electrolytes into cells, leading to hypophosphatemia, hypokalemia, and hypomagnesemia, that can have significant cardiac consequences (9, 27). Electrolyte derangements, whether from re-feeding or the underlying eating disorder itself, are well-known contributors to QTc interval prolongation, as they impair cardiac repolarization by disrupting ion channel function (28, 29). Additionally, psychotropic and non-psychotropic medications commonly used in eating disorder management can further exacerbate this risk, either by directly affecting ion channels or indirectly causing electrolyte imbalances (29, 30). Given the ongoing debate on the intrinsic role of eating disorders in QTc prolongation, identifying the most significant risk factors which can contribute to QTc prolongation and cardiac death is crucial. This study seeks to clarify the impact of extrinsic factors, specifically QTc-prolonging medication use and electrolyte imbalances, on QTc interval prolongation in pediatric patients with eating disorders.

## 2 Materials and methods

### 2.1 Patients

Approval for this study was granted by the Hamilton Integrated Research Ethics Board (HiREB). A complete retrospective chart analysis was conducted for 435 patients between the ages of 6 and 17. Electronic medical records on EPIC were reviewed to collect demographic information such as sex assigned at birth, age, and date of hospital visit. All patients were assessed by the McMaster Children's Hospital Eating Disorder Program in Hamilton, Ontario, Canada, between January 9, 2019 and December 9, 2023. Patients were evaluated in a variety of settings, including the outpatient Eating Disorders clinic, emergency department, and inpatient units. Eligibility criteria for inclusion required that patients had a confirmed eating disorder diagnosis and were under 18 years of age at the time of the clinical encounter. All patients were required to have at least one ECG recorded during their hospital visit which had been interpreted by a pediatric cardiologist and was uploaded to the medical record with sufficient resolution for further interpretation. Within 72 h of the ECG, patients were also required to have non-hemolyzed serum levels of calcium, magnesium, and potassium. Patients with known cardiac diagnoses or congenital electrolyte abnormalities were excluded from the study due to possible influence on the QTc interval. Finally, any ECGs with right bundle branch block, T-wave abnormalities (such as flat T wave), ST segment changes, or baseline artifacts were not eligible for the study due to their confounding effect on QTc interval calculation.

### 2.2 ECG assessments

ECGs were reviewed to document the machine-calculated heart rate and Bazett QTc interval, reported as QTc-M. Commentary from the pediatric cardiologist reviewing the ECG was also documented. In addition, QTc intervals were manually calculated by one of four interpreters using the Bazett formula (QTc = QT/√RR) and reported as QTc-B. However, a known limitation of the Bazett formula is its tendency to overestimate the QTc at lower heart rates (31). For patients with profound bradycardia, defined by age-based ranges (32), interpreters also used the Fridericia formula (QTc = QT/∛RR) to calculate the QTc, reported as QTc-F. For each manual QTc calculation, the QT interval and the preceding RR interval were measured from lead V5 of a standard 12-lead ECG. If the intervals could not be clearly identified in lead V5, then lead II was used. The QT interval was measured from the onset of the Q wave (the transition from the isoelectric point to negative deflection) to the termination of the T wave (the point at which the terminal limb of the T-wave intersects the isoelectric line). Importantly, this point was estimated using the slope of the T-wave and did not include the U wave. The RR interval was calculated starting at the R-wave preceding the QRS complex used to calculate the QTc interval. A normal QTc value was defined as <440 milliseconds, a borderline QTc value as ≥440 and <460 milliseconds, and a prolonged QTc value as >460 milliseconds (33).

In ECGs with sinus arrhythmia, the QT and RR intervals were measured from lead II, using the most frequently occurring RR interval, which was deemed the most representative RR interval. Two methods were used to identify which QRS complex associated with the representative RR interval would be assessed. If there were sequential or representative RR intervals in series, the last RR interval was selected in this series before the resumption of sinus arrhythmia. If no series was identified and the representative RR intervals were dispersed, the first representative RR interval was selected.

### 2.3 Clinical data

Electronic medical records were reviewed to collect laboratory data and relevant clinical information. Serum electrolyte levels of calcium, magnesium, and potassium were obtained from blood work performed closest to the time of ECG collection, within a 72-hour window. Normal serum electrolyte ranges were defined as calcium within 2.22–2.62 mmol/L, magnesium within 0.70–0.91 mmol/L, and potassium within 3.5–5.2 mmol/L. Clinical notes from the Eating Disorder Program were reviewed to determine the eating disorder diagnosis, setting in which the patient was assessed, and whether the patient was taking any medications known to prolong the QTc interval at the time of the encounter. Medications that may prolong the QTc interval were documented if they were associated with a known, possible, or conditional risk of causing QT prolongation and TdP, based on the QTdrugs List created by the Arizona Center for Education and Research on Therapeutics (34). Finally, subsequent notes were reviewed to document any changes to management on the basis of a prolonged QTc interval and whether the patient sustained ventricular tachycardia, torsades de pointes, or sudden cardiac death.

### 2.4 Statistical analysis

Normal serum electrolyte ranges were defined by the laboratory at McMaster Children's Hospital and analyzed as both numerical and categorical (low, normal, high) variables. Machine calculated Bazett QTc (QTc-M), manually calculated Bazett QTc (QTc-B) and manually calculated Fridericia QTc (QTc-F) were determined according to procedures discussed above and analyzed as both numerical and categorical (normal, borderline, prolonged) variables. Eating disorder diagnosis (AN, BN, ARFID, OSFED, BED, rumination) and QTc-prolonging medication use (none, one, multiple), care setting (inpatient, outpatient), and sex were also analyzed as categorical variables.

Fisher's exact test was used to determine the independence of the QTc-B category and sex, eating disorder diagnosis, serum electrolyte abnormalities, care setting, and QTc-prolonging medication use, respectively. The independence of average numerical QTc-B and sex, care setting, and QTc-prolonging medication use, respectively, were assessed using either Welch's unpooled 2-sample *t*-test or Student's pooled *t*-test, with the assumption of equal variances determined using the F-statistic. ANOVA was used to assess the independence of average numerical QTc-B and eating disorder diagnosis and electrolyte abnormalities, respectively. Both Pearson's product-moment correlation and Spearman's rank correlation were used to assess the correlation between serum electrolyte numerical values and QTc-B.

To evaluate the interrater reliability of the four QTc-B assessors, the intraclass correlation coefficient (ICC) was assessed across a subset of 35 random participants prior to any further analysis. The ICC was also used to compare agreement between QTc-B and QTc-M calculations, as well as QTc-B and QTc-F in patients with bradycardia. Statistical analysis of patient data was performed using the R software platform. A *p*-value of <0.05 was considered statistically significant in all analyses.

## 3 Results

### 3.1 Initial study population

A comprehensive patient list for the years 2019–2023 was provided by McMaster Children's Hospital Eating Disorder Program for analysis. Of the 947 patients on the initial list, over half of the individuals (54.07% *n* = 512) did not meet the study's inclusion criteria upon further screening. Approximately 14% (*n* = 71) of these ineligible patients were excluded as they were not diagnosed with an eating disorder despite assessment by the Eating Disorder team. The most common exclusion criteria was a lack of ECG uploaded to the chart or an ECG performed outside of childhood (*n* = 298). Of the remaining patients with ECGs, 50 were excluded due to poor digital ECG quality which could not be read for analysis. Another 75 patients did not have electrolytes collected within 72 h of their ECG or were missing at least one of the required electrolytes. Incomplete clinician documentation, including an absent medication history, lead to another 6 patients being excluded. A total of 5 patients were excluded due to confounding medical diagnoses, including congenital electrolyte abnormalities, history of recurrent arrhythmias, myocarditis, congenital heart disease, and Wolff–Parkinson–White syndrome. A final 7 patients were excluded due to ECG abnormalities impacting the T-wave or ECG baseline.

### 3.2 Reliability of QTc-B calculations

As the values of manual QTc were calculated by four independent assessors, the intraclass correlation coefficient (ICC) using a one-way random effects model was used to assess consistency across an initial subset of 35 participants. This was performed to evaluate reliability for the study's primary outcome measure of manual QTc Bazett calculation (QTc-B). The ICC for QTc-B was 0.925 (95% CI: 0.874–0.958), indicating excellent agreement among raters. The ICC was also evaluated for the measured values of QT and RR across the four assessors. The correlations for QT and RR measurements were 0.944 (95% CI: 0.907–0.969), and 0.990 (95% CI: 0.983–0.994), respectively, both reflecting high consistency in measurement. These results suggest that manual QTc-B calculations were highly reproducible among assessors, supporting the reliability of manually calculated QTc-B values in this study.

### 3.3 Demographics

A total of 435 patients were eligible for inclusion in the study with an average age of 14.22 (± 1.97) and an age range of 6–17. The majority of patients were female (86.4% *n* = 376) and over half of all patients 57.7% received care in the outpatient setting (*n* = 251). A subset of patients (37.5% *n* = 163) were taking one or more medications known to prolong the QTc interval. The most prevalent diagnosis was Anorexia Nervosa (AN) (57.24%, *n* = 249) followed by Avoidant Restrictive Food Intake Disorder (20.23%, *n* = 88) and Other Specified Feeding and Eating Disorder (16.09%, *n* = 70). Further patient demographics are outlined in Table 1.

**Table 1:** Baseline characteristics of this study's population of 435 patients with eating disorders.

Baseline Demographics	
Age (years)	14.22 ± 1.97
Female	376 (86.44%)
Outpatient	251 (57.70%)
QTc-prolonging medication use	163 (37.47%)
One QTc-prolonging medication	119 (73.00%)
Two or more QTc-prolonging medications	44 (26.99%)
Eating Disorder Type Prevalence	
Anorexia Nervosa	249 (57.24%)
Bulimia Nervosa	20 (4.60%)
ARFID	88 (20.23%)
OSFED	70 (16.09%)
BED	7 (1.61%)
Rumination	1 (0.23%)

ARFID, avoidant restrictive food intake disorder; OSFED, other specified feeding or eating disorder; BED, binge-eating disorder.

Assessment of ECGs for the study population demonstrated a normal average heart rate not suggestive of bradycardia (66.91 ± 15.79 ms). The average automatic machine QTc interval (QTc-M), manual Bazett (QTc-B), and manual Friderica (QTc-F) intervals were all within normal limits. Additionally, the prevalence of prolonged QTc using QTc-B was 5.06% of the population as compared to 2.07% using QTc-M. Further average population ECG parameters are demonstrated in Table 2.

**Table 2:** Mean population ECG parameters across 435 eligible patients.

ECG parameter	**Value**
HR (bpm)	66.91 ± 15.79
RR (s)	0.94 ± 0.24
QT-M (s)	0.38 ± 0.04
QTc-M (ms)	411.22 ± 26.09
Normal	406 (93.33%)
Borderline	20 (4.60%)
High	9 (2.07%)
QTc-B (ms)	400.83 ± 33.34
Normal	405 (93.10%)
Borderline	8 (1.84%)
High	22 (5.06%)
QTc-F (ms)^a^	395.65 ± 31.15

HR, heart rate; RR, RR interval; QT-M, machine-calculated QT interval; QTc-M, machine-calculated QTc interval; QTc-B, QTc interval, manual Bazett formula; QTc-F, QTc interval, manual Friderica formula.

a Subset of *n* = 26 patients.

### 3.4 Comparison of QTc-B among demographic subgroups

Subgroup analyses were conducted to compare mean QTc-B values between sexes, setting of care delivery, and across eating disorder subtypes. Within both male and female subgroups, the mean QTc-B was within the normal range, although the mean QTc-B was significantly longer in females (402.82 ± 32.84 ms vs. 388.16 ± 34.02 ms, *p* = 0.0016). Borderline QTc-B intervals were observed in 2.1% of females but not observed in any males, while prolonged QTc-B intervals were seen in 5.3% of females and 3.4% of males. Differences in the prevalence of borderline and prolonged QTc-B between male and female participants did not reach statistical significance (*p* = 0.662).

There was no significant difference in mean QTc-B between inpatients (397.32 ± 36.08 ms) and outpatients (403.40 ± 31.01 ms, *p* = 0.066). The prevalence of borderline QTc-B intervals was 1.6% in inpatients and 2.0% in outpatients, while prolonged QTc-B was observed in 4.9% of inpatients and 5.2% of outpatients. There was no significant association between setting of care delivery and prevalence QTc-B interval prolongation (*p* = 1.0).

**Figure 1:**
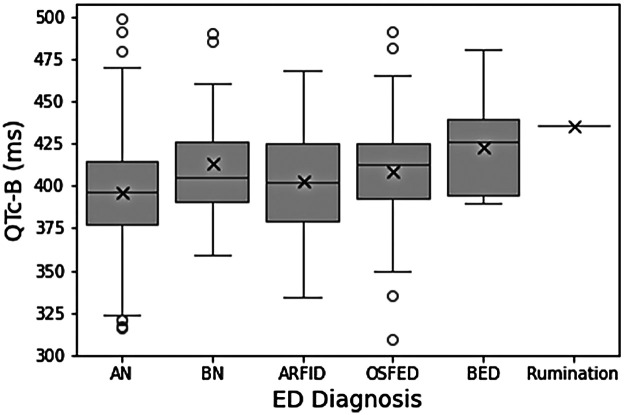
QTc-B interval lengths across represented eating disorder diagnoses.×represents mean values, horizontal lines indicate inclusive medians, and circles represent outlier values. ED, eating disorder; QTc-B, QTc interval, manual Bazett formula; AN, anorexia nervosa; BN, bulimia nervosa; ARFID, avoidant/restrictive food intake disorder; OSFED, other specified feeding or eating disorder; BED, binge-eating disorder.

Mean QTc-B values varied significantly across eating disorder subtypes (*p* = 0.0063). Patients with bulimia nervosa (BN) and binge-eating disorder (BED) exhibited the longest QTc-B values (BN: 413.55 ± 36.19 ms, BED: 423.47 ± 33.53 ms), while those with AN had the lowest mean QTc-B (396.18 ± 33.08 ms). The prevalence of prolonged QTc-B was more frequent in BN (15.0%) and BED (14.3%) when compared to AN (3.6%) (*p* = 0.3616). However, investigators note the small sample sizes for certain subgroups including both BED (*n* = 7) and Rumination Disorder (*n* = 1) (Figure 1).

### 3.5 Bradycardia

Although 409 patients had normal or elevated heart rates, a total of 26 patients met the criteria for profound bradycardia based on their age. The average QTc-B was 402.7 ± 32.7 ms for patients without bradycardia and 372.2 ± 29.9 ms in those with bradycardia. Importantly, all patients with bradycardia had normal QTc intervals using the Bazett formula, though two of these patients were classified as prolonged when the Fridericia formula was applied. Overall, though there was good agreement between these two methods of calculation, the agreement did not meet the threshold of statistical significance (ICC = 0.744, *p* = 0.0623—see Supplementary data). There was no significant association between bradycardia status and whether patients had QTc-B classified as normal, borderline or prolonged (*p* = 0.6256).

### 3.6 QTc-Prolonging medications

**Figure 2:**
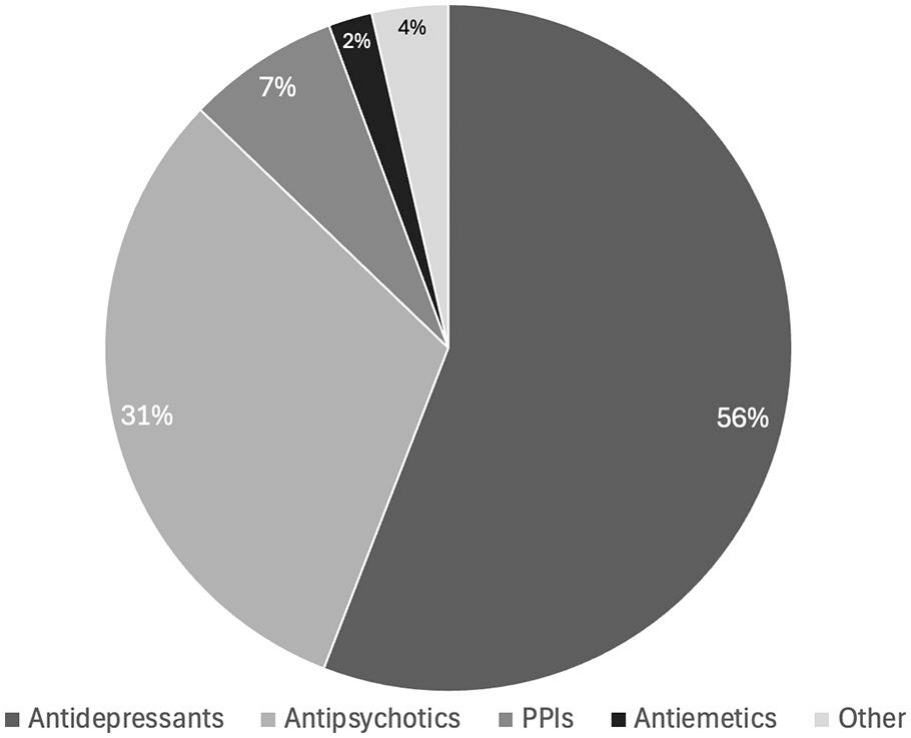
General classification of QTc-prolonging medications used by patients.

The majority of patients taking known QTc-prolonging medications were only taking a single agent (73.01% *n* = 119) while the remaining 26.99% were taking two or more medications. Upon review, 26 unique medications (*n* = 195) were documented with psychotropics as the most frequently used medications (89.70%) as demonstrated in Figure 2. Antidepressants (*n* = 109), primarily selective serotonin reuptake inhibitors, accounted for the majority of psychotropic use with fluoxetine identified as the most common agent (*n* = 56). Other antidepressants included escitalopram (*n* = 15), sertraline (*n* = 13), citalopram (*n* = 9), venlafaxine (*n* = 8), trazodone (*n* = 3), fluvoxamine (*n* = 2), mirtazapine (*n* = 2), and bupropion (*n* = 1). Antipsychotics (*n* = 61), primarily second generation, were the second most common psychotropic class used, and olanzapine was the most frequently used atypical antipsychotic (*n* = 31). In the same class, quetiapine (*n* = 15), risperidone (*n* = 9), aripiprazole (*n* = 5), and perphenazine (*n* = 1) were also used.

Other medications included proton-pump inhibitors (*n* = 14) and antiemetics (*n* = 4). Of the proton-pump inhibitors, lansoprazole was the most prescribed (*n* = 6) followed by pantoprazole, omeprazole, and rabeprazole. Antiemetics included domperidone (*n* = 2), metoclopramide (*n* = 1), and ondansetron (*n* = 1). Finally, other agents consisted of atomoxetine (*n* = 2), leuprolide (*n* = 2), hydroxychloroquine (*n* = 1), lamotrigine (*n* = 1), and lisdexamfetamine (*n* = 1).

**Figure 3:**
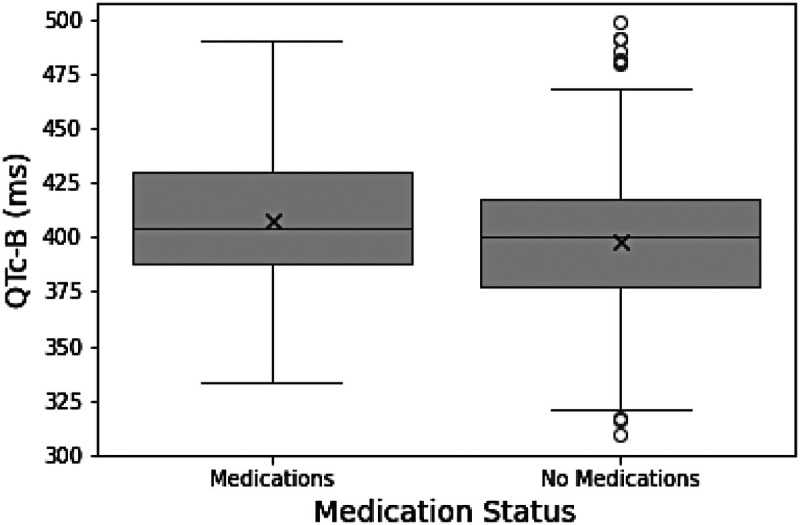
QTc-B interval lengths according to use of QTc-prolonging medications. X represents the mean values of 408.84 ms for those taking such medications and 396.03 ms for those not (*p* < 0.001). Horizontal lines indicate the inclusive medians for these respective groups. Outlier values are represented by a circle. QTc-B, QTc interval, manual Bazett formula.

The mean QTc-B interval was significantly longer in patients taking at least one QTc-prolonging medication (*n* = 163) compared to patients who were not taking any of these medications (*n* = 272) (408.84 ms vs. 396.03 ms, *p* < 0.001) (Figure 3). However, there was no significant association between medication use and distribution of QTc-B length as normal, borderline or prolonged (*p* = 0.145). Prolonged QTc-B occurred in 4.04% (*n* = 11) of patients not on medications and 6.75% (*n* = 11) of patients on QTc-prolonging medications. For both groups, normal QTc-B interval length was predominant (94.85% vs. 90.18%, respectively).

The mean QTc-B interval was not significantly different between patients taking one medication (*n* = 119, 406.97 ms) and those taking two or more medications (*n* = 44, 411.18 ms, *p* = 0.568). In addition, there was no statistically significant association between taking more than one medication and distribution across the different categories of QTc-B length (*p* = 0.207). The prevalence of prolonged QTc-B was 6.72% (*n* = 8) in the single medication group and 6.82% (*n* = 3) in the multiple medications group. Classification of the QTc-B interval in patients taking medications known to prolong the QTc interval is further outlined in Table 3.

**Table 3:** Classification of QTc-B interval according to use of QTc-prolonging medications.

QTc-prolonging medication use	QTc normal	QTc borderline	QTc prolonged	***p*-value**
No Medications	258	3	11	*p* = 0.1450
Medications	147	5	11	
One Medication	108	3	8	*p* = 0.2071
Multiple Medications	39	2	3	

QTc Normal: <440 ms; QTc Borderline: 440–460 ms; QTc Prolonged: >460 ms (31). QTc-B, QTc interval, manual Bazett formula.

### 3.7 Electrolytes

As presented in Table 4, the majority of patients had normal levels of potassium, calcium, and magnesium. For all patients, average serum electrolyte values for calcium (2.39 ± 0.11 mmol/L), magnesium (0.844 ± 0.075 mmol/L), and potassium (4.00 ± 0.37 mmol/L) were also within the normal clinical ranges. Most patients (*n* = 405) had normal calcium values while 5 were hypercalcemic and 25 were hypocalcemic. The classification of calcium levels into normal, high, or low was independent of categorical QTc-B classification (*p* = 0.4884) and there was no difference in average QTc-B values across these three groups (*p* = 0.1235) as shown in Table 5.

**Table 4:** Comparison of categorical QTc-B classification according to potassium, calcium, and magnesium abnormalities.

**Electrolyte**	**QTc normal**	**QTc borderline**	**QTc prolonged**	***p*-value**
Potassium				
Low	18	0	3	*p* = 0.2481
Normal	385	8	19	
High	2	0	0	
Calcium				
Low	22	1	2	*p* = 0.4884
Normal	378	7	20	
High	5	0	0	
Magnesium				
Low	5	0	1	*p* = 0.4262
Normal	340	8	18	
High	60	0	3	

Normal serum electrolyte ranges are defined as Ca^2+^: 2.22–2.62 mmol/L, Mg^2+^: 0.70–0.91 mmol/L; K^+^: 3.5–5.2 mmol/L. QTc Normal: <440 ms; QTc Borderline: 440–460 ms; QTc Prolonged: >460 ms (31)**.** QTc-B, QTc interval, manual Bazett formula.

**Table 5:** Comparison of mean QTc-B values according to potassium, calcium, and magnesium abnormalities.

Electrolyte	**Low**	**Normal**	**High**	*p*-value
Potassium:				*p* = 0.8824
*N* (%)	21 (4.83%)	412 (94.71%)	2 (0.46%)	
Mean QTc-B (ms)	404.25 ± 36.76	400.64 ± 33.2	403.98 ± 46.08	
Calcium:				*p* = 0.1235
*N* (%)	25 (5.75%)	405 (93.10%)	5 (1.15%)	
Mean QTc-B (ms)	406.52 ± 36.96	400.83 ± 33.03	373.11 ± 33.12	
Magnesium:				***p*** **=** **0.0323**
*N* (%)	6 (1.38%)	366 (84.14%)	63 (14.48%)	
Mean QTc-B (ms)	413.55 ± 43.21	402.28 ± 32.26	391.18 ± 37.20	

Normal serum electrolyte ranges are defined as Ca^2+^: 2.22–2.62 mmol/L, Mg^2+^: 0.70–0.91 mmol/L; K^+^: 3.5–5.2 mmol/L. QTc-B, QTc interval, manual Bazett formula.Bold values indicate statistical significance (*p* < 0.05).

Most patients also had a normal magnesium level (*n* = 366), but a larger proportion had hypermagnesemia (*n* = 63) rather than hypomagnesemia (*n* = 6) relative to the two other electrolytes studied. Categorical QTc-B classification and magnesium classification were found to be independent (*p* = 0.4262) (Table 4). Average QTc-B values were significantly different for patients with hypomagnesemia, normal magnesium levels, and hypermagnesemia (*p* = 0.03232) (Table 5). Importantly, QTc-B was longest in patients with low magnesium and shortest in patients with high magnesium.

Nearly 95% of patients had normal potassium values (*n* = 412). Of the remaining 5%, 21 were hypokalemic and only 2 were hyperkalemic. Classification of potassium into low, normal, or high was also independent of categorical QTc-B classification (*p* = 0.2481) (Table 4) and there was no difference in average QTc-B value across the three groups (*p* = 0.8824) (Table 5).

Patients with normal, borderline, and prolonged QTc-B intervals had no difference in average calcium, magnesium, or potassium levels (*p* = 0.06615, *p* = 0.1729, *p* = 0.3397). Despite this, there was a negative correlation between electrolyte levels and QTc-B interval length such that higher QTc-B values were associated with lower values of calcium (Pearson correlation −0.157; *p* = 0.00014, Spearman correlation −0.142; *p* = 0.00058), magnesium (Pearson correlation −0.107; *p* = 0.0096, Spearman correlation −0.113; *p* = 0.006031), and potassium (Pearson correlation −0.124; *p* = 0.00273, Spearman correlation −0.103; *p* = 0.01296).

### 3.8 Adverse cardiac events

Importantly, there were no documented occurrences of ventricular tachycardia, torsades de pointes, or sudden cardiac death during the study period. Additionally, for those with prolonged QTc intervals, no interventions were documented regarding substantive changes in management. The only consultations to pediatric cardiology occurred due to pericardial effusion, suspected left ventricular hypertrophy, sinus bradycardia with early repolarization, and suspected ischemia with associated dyspnea and chest pain. All of the consultations were followed with echocardiograms, Holter monitoring and/or exercise tests which ultimately did not result in any changes to medical management. Finally, several patients had multiple electrocardiograms performed during the study period. The longitudinal ECGs which met the inclusion criteria were of insufficient numbers, had significant temporal variation, and were influenced by other confounding factors such as changes to QTc-prolonging medications which did not enable a fulsome subgroup analysis.

## 4 Discussion

Within this diverse cohort of pediatric eating disorder patients, only 6.9% (*n* = 30) demonstrated borderline or prolonged QTc intervals using the widely clinically applied Bazett formula. Although this prevalence is reassuringly low, there are no current figures in the literature to estimate the baseline prevalence of QTc prolongation in a healthy pediatric population which limits the generalizability of this statistic. Importantly, in the minority of eating disorder patients with borderline or prolonged QTc-B intervals, none had an associated arrhythmia or major adverse cardiac event. Specifically, no patients had documented torsades de pointes or sudden cardiac death in the context of QTc-B prolongation.

When compared to articles from the last decade examining QTc interval in pediatric patients with eating disorders, the current findings contribute to the growing body of evidence suggesting a lack of inherent QTc prolongation as shown in Table 6. A particular strength of this study is the analysis of participant subgroups including sex, all QTc prolonging medications, and individual eating disorder diagnosis. This study's large sample size, assessment of care setting, and the cohort of exclusively pediatric patients are also notable distinctions from the prior literature.

**Table 6:** Selected studies from 2016 to 2026 assessing QTc interval in predominately pediatric patients with eating disorders.

Article	Age**s**	Sex	ED **included**	**QTc prolonging m**edications	**Mean Bazett QTc (ms)**	Prolonged QTc** > 460 ms *n* (%)**
Benayon et al. Canada (19)	7–17	Mixed	Various, not specified	Psychotropics only	411.0 ± 25.9	11 (4.2%)
Bomba et al. Italy (35)	13–17	Female	AN	None	386.6 ± 26.3	None
Choi et al. Korea (36)	10–18	Mixed	Various, not specified	No drugs affecting cardiovascular system	Not provided	12 (9.4%)
Dinardo et al. USA (37)	8–23	Mixed	AN, ARFID, BN, EDNOS	Included	400	5 (2.5%)^a^
Green et al. USA (38)	14–38	Female	AN, BED, BN, OSFED	Not identified	AN: 390 ± 30	Not identified
					BED: 390 ± 20	
					BN: 390 ± 20	
					OFSED: 390 ± 30	
Guerrier et al. USA (15)	11–25	Female	AN	Included	429.0 ± 37.9	8 (9.8%)^b^
Janzen et al. Canada (20)	15 ± 2	Female	AN	Psychotropics only	No medication: 401 ± 24	1 (3%)
					Medication: 407 ± 26	
Letizia et al. Italy (23)	10–18	Mixed	AN, ARFID, BED, BN, UFED	Psychotropics only	406.7 ± 22.9	None
Current study	6–17	Mixed	AN, ARFID, BED, BN, OSFED, Rumination	Included	400.83 ± 33.34	22 (5.06%)

QTc, corrected QT interval; ED, eating disorder; AN, anorexia nervosa; ARFID, avoidant/restrictive food intake disorder; BN,bulimia nervosa; EDNOS, eating disorder not otherwise specified; BED, binge-eating disorder; OSFED, other specified feeding or eating disorder; UFED, Unspecified Feeding or Eating Disorder.

a Criteria of >450 ms for <18 years and >460 ms for >18 years.

b Criteria of >440 ms.

Relative to adult male eating disorder populations, pediatric males represent a higher proportion of those seeking eating disorder treatment but remain an understudied group in the literature (39). Among the few studies analyzing QTc intervals in mixed sex pediatric patients with eating disorders, most patients did not have prolonged QTc intervals (15, 17, 19, 21, 23, 36). Our study supports the lack of QTc-B prolongation in patients with eating disorders for both sexes. Despite both sexes having QTc-B averages within normal clinical limits, we found longer mean intervals in female participants (402.82 ± 32.84 ms vs. 388.16 ± 34.02 ms, *p* = 0.0016). There is an inherent sex-based difference in QTc intervals after puberty with post-pubertal females considered to be at increased risk of developing arrhythmias with prolonged QTc intervals (40, 41). The underlying mechanism remains unclear but may be associated with pubertal hormonal changes (41, 42).

To our knowledge, there are no previous studies directly comparing QTc intervals between outpatient and inpatient pediatric eating disorder populations. Our analysis found no significant differences in mean QTc-B intervals between inpatients (397.32 ± 36.08 ms) and outpatients (403.40 ± 31.01 ms, *p* = 0.066) and no association between QTc-B interval length and care delivery setting (*p* = 1.0). Moreover, the prevalence of borderline QTc-B intervals was similarly low in both groups (1.6% in inpatients and 2.0% in outpatients), and the proportion of patients with prolonged QTc-B intervals was comparable (4.9% in inpatients and 5.2% in outpatients). These results suggest that care setting was independent of QTc-B interval length in this cohort despite cardiac risk factors, such as bradycardia and hypotension, which can serve as possible indications of medical instability for admission.

In the analysis of individual eating disorder diagnoses, the average QTc-B interval of patients with AN was within normal limits (396.18 ± 33.08 ms). Importantly, AN was the most common eating disorder diagnosis represented in our study and the prevalence of QTc-B prolongation was low in this group. Multiple adolescent studies also demonstrated longer QTc intervals in healthy controls or patients with other eating disorders relative to AN (17, 35, 37). This finding juxtaposes the proposed inherent QTc prolongation in AN which has been suspected to result from myocardial ischemia, altered myocardial recovery, reduced left ventricular mass, and smaller cardiac chamber size induced by starvation states (14, 18, 22).

Although our study demonstrated the longest QTc-B interval in BED (423.47 ± 33.53 ms), this subgroup had a small number of patients (*n* = 7) which limits the statistical power of these findings. Additionally, there is a lack of substantive evidence in the literature regarding QTc intervals in pediatric BED. Furthermore, this eating disorder is not as prevalent or well-studied as AN. Patients with BN had the second longest QTc-B interval (413.55 ± 36.19 ms) which has also been previously reported in the literature (17). However, findings in BN populations remain mixed, as other adolescent studies have reported either shortened or borderline prolonged QTc intervals in BN (37, 43). Less evidence exists for QTc prolongation in ARFID, OFSED, and rumination. However, there is some literature that suggests adolescent patients with ARFID and OFSED present with QTc intervals within normal limits and which are comparable to those observed in patients with AN (15, 37, 38).

In our study of 435 patients, 26 met criteria for profound bradycardia (32). Although the literature suggests that bradycardia can prolong repolarization, our cohort patients with bradycardia had shorter average QTc-B intervals compared to those with normal or even elevated heart rates (372.2 ± 29.9 ms vs. 402.7 ± 32.7 ms) (40). It is well recognized that the Bazett formula, commonly used in clinical practice, tends to overestimate QTc values in patients with low heart rates due to its square-root correction of the RR interval (44). In contrast, the Fridericia formula applies a less aggressive correction and may offer greater accuracy in bradycardic populations, such as individuals with eating disorders. In our cohort, all bradycardic patients had QTc-B intervals within the normal range, and only two patients demonstrated QTc-F prolongation when corrected using the Fridericia formula. These findings, consistent across both correction methods, suggest that bradycardia alone was not significantly associated with clinical QTc prolongation in this population. Agreement between the Bazett and Fridericia formulas was moderate to good [intraclass correlation coefficient (ICC) = 0.744], though this was not statistically significant and extrapolations may be limited due to small sample size of patients with profound bradycardia. Given that bradycardia in the context of eating disorders is one of many physiologic adaptations to malnutrition, our findings indicate that isolated bradycardia may not be associated with prolonged QTc duration unless additional risk factors are present.

QTc-prolonging medications are a known risk factor for QTc interval prolongation. The average QTc-B interval was found to be longer in patients taking at least one QTc-prolonging medication, compared to patients who were not taking such medications (408.84 ms vs. 396.03 ms, *p* < 0.001). Despite evidence in the literature suggesting otherwise, our study found no difference in the average QTc-B interval when comparing patients taking only one medication relative to those taking multiple QTc-prolonging agents (*p* = 0.568) (35, 41). QTc-prolonging medications are categorized by their risk of causing QTc prolongation as either known, possible, or conditional risk by the QTc drugs list (34). Importantly, for the same reported categorization, the strength of associated evidence, proposed mechanism of QTc prolongation, and reported adverse effects vary widely (34, 40, 45, 46). This study used any medication identified within the QTc drug database, including those with conditional risk, due to the likelihood of additional risk factors in an eating disorder population, including electrolyte abnormalities and possible substance use (41, 45, 46).

Across the three electrolytes examined, there was no statistically significant difference in QTc-B interval classification based on whether the serum electrolytes were categorized as low, normal or high. Despite the lack of consistent group-level differences, there was a significant negative correlation between levels of all three electrolytes: calcium (Pearson *r* = −0.157; *p* = 0.00014), potassium (*r* = −0.124; *p* = 0.00273), and magnesium (*r* = −0.107; *p* = 0.0096) and QTc-B interval. These findings suggest that lower electrolyte values were associated with longer QTc-B intervals, even within clinically normal ranges. This trend aligns with previous literature demonstrating that low levels of potassium, calcium, and magnesium can contribute to QTc prolongation. However, these effects are generally magnitude-dependent and most pronounced at more severe levels of deficiency (47–49). Previous studies have shown that QTc prolongation is more likely to occur when potassium levels fall below 3.0 mmol/L, a threshold not reached by any patients in our cohort which is important when interpreting hypokalemia findings in this group (47). While hypokalemia has been frequently observed in adult patients with markedly prolonged QTc intervals (24), the average QTc-B was not significantly longer for patients with hypokalemia in our study. Hypocalcemia is known to prolong the QTc interval by disrupting calcium-dependent ion channel function (48), and similarly, the degree of prolongation correlates with the severity of hypocalcemia (49). Despite this, the mean QTc-B was similar across those with normal and low levels of serum calcium in our cohort.

Magnesium was the only electrolyte to show a statistically significant difference in mean QTc-B interval between the classification of low, normal, and high magnesium levels (*p* = 0.0323). The mean QTc-B interval for hypomagnesemic patients was significantly higher than patients with normal or elevated serum magnesium although this finding is limited due to small sample size of patients with hypomagnesemia (*n* = 6). Prior research supports a link between low magnesium and QTc prolongation, with studies showing changes in ventricular repolarization and QTc duration which normalize upon magnesium repletion (50). Overall, these findings suggest that although electrolyte abnormalities are mechanistically linked to QTc prolongation, the strength of this relationship is likely dependent on the severity of the imbalance. As the classification of patients with abnormal electrolytes was not stratified by the magnitude of the disturbance, patients were classified as abnormal based on differences as small as 0.01 mmol/L from the accepted reference range. Further, the rarity of clinically significant or profound electrolyte disturbances in this cohort limited our ability to detect differences across classification groups. However, the consistent negative correlations observed indicate that even mild reductions in serum electrolyte levels may influence QTc-B prolongation.

### 4.1 Limitations

This study aimed to determine the relationship between eating disorder diagnoses and prolonged QTc interval in the pediatric population. As an observational and retrospective study, a lack of standardized data collection may have introduced unknown confounding factors to the analysis. As a single-centre study including patients treated at a tertiary pediatric academic hospital, the generalizability of study results to the broader population of pediatric patients with eating disorders may be limited as community data was not represented and findings may be influenced by site specific practices. Furthermore, there was a large number of eating disorder patients within the study population who were excluded from analysis due to factors including a lack of ECG or electrolyte samples. This exclusion introduces the potential for bias in included subjects. External validity is further limited by the heterogeneity of eating disorder presentations included as there is a wide spectrum of disease chronicity and morbidity not adequately captured by our analysis. A lack of available data on baseline QTc prior to eating disorder diagnosis and concurrent genetic risk factors also limits these findings.

As discussed above, serum electrolyte values were included if all three (potassium, calcium, magnesium) were recorded within 72 h of a participant's ECG to reduce temporal variability. However, the range of time intervals between ECG and electrolyte measurements make it challenging to establish definitive relationships between these factors. Moreover, participants lacking all three electrolyte values within 72 h of an ECG were excluded which limits sample size and introduces selection bias. Additionally, investigators did not evaluate differences in magnitude of electrolytes such as severely abnormal or critical samples due to low sample size. Further, assessors did not collect data on medication adherence, dosing, or timing, preventing evaluation of dose-dependent effects. Additionally, as no distinction was made between the types of QTc-prolonging medications used, differences in drug-specific effects on QTc were not considered in the analysis.

Some potential confounding factors in the data are a lack of adjustment for refeeding, hydration status, body mass index, substance use, and misuse of diuretics or laxatives, all of which could have influenced both QTc and serum electrolytes. QTc cutoffs were not corrected for age, sex, or pubertal status as discussed previously in the literature (33, 42, 51), ignoring a potentially significant source of biological variability. However, it should be noted that there is significant disagreement in the literature regarding how to correct QTc ranges for age. Due to the retrospective nature of this study, Tanner staging to determine pubertal onset was not feasible and utilizing age-based cut-offs is debated in the literature. Further, several participants were concurrently undergoing gender affirming therapies and thus pubertal onset would not have been an accurate measure in these individuals.

Finally, this study does have important limitations regarding statistical power. The large number of subgroup analyses performed with the data increases the risk of Type I error and should lead to these results being interpreted as exploratory in nature. Additionally, the prevalence of borderline or prolonged QTc-B in the participants was low (6.9%), and even lower for the identified subgroups. Investigators therefore likely failed to establish several significant associations among the categorical variables due to under-sampling of the population. Similarly, small population sizes limited statistical findings in patients with abnormal serum electrolytes, less common eating disorder subtypes (BED and rumination), and those prescribed multiple QTc-prolonging medications. The findings from this study are likely subject to confounding variables due to the lack of multivariable analyses performed. More fulsome analysis of these relationships in the future would require a far larger sample, given the low incidence of QTc abnormalities in the population under study, and thoughtful consideration of these several limitations.

## 5 Conclusion

This study demonstrates the multifactorial nature of QTc interval prolongation in children and adolescents with eating disorders. It is crucial for clinicians to identify and address secondary causes for QTc prolongation in this population rather than concluding that an eating disorder diagnosis alone is responsible for this finding. Specifically, QTc-prolonging medications and low serum electrolytes were associated with QTc-B interval prolongation in this cohort. Across the eating disorders assessed, patients with AN demonstrated the shortest QTc-B intervals. The notable differences in mean QTc-B across eating disorder subtypes warrant further investigation to elucidate contributing factors which may be used to identify patients at higher risk of QTc interval prolongation. These results also highlight the importance of monitoring baseline QTc intervals in patients with any eating disorder diagnosis due to the prevalence of risk factors, including electrolyte abnormalities and QTc-prolonging medications, in this population. Further research could benefit from larger sample sizes to better capture other factors impacting QTc interval prolongation and explore longitudinal patient outcomes as some ECG changes can be reversed with recovery (36).

## Data Availability

The raw data supporting the conclusions of this article will be made available by the authors, without undue reservation.
