# Supplementary material for: QTc interval prolongation in pediatric eating disorder population
Source: Front Pediatr. 2026 Jun 26;14:1854024. doi: 10.3389/fped.2026.1854024 (PMC13349899; doi:10.3389/fped.2026.1854024)
Supplement: Supplementary file 1 [file Supplementaryfile1.docx]

# Supplemental Data

## Correlation Between Machine and Manual QTc-Bazett Calculations

To assess the agreement between machine-calculated and manually measured QTc-Bazett (QTc-B) values, we conducted Intraclass Correlation Coefficient (ICC), Cohen’s Kappa, Pearson’s correlation, and Spearman’s correlation analyses. The ICC for absolute agreement between machine and manual QTc-B was 0.723 (95% CI: 0.559–0.815, p < 0.0001), indicating moderate reliability. Cohen’s Kappa for categorical agreement of QTc classification (normal, borderline, prolonged) was 0.48 (unweighted) and 0.65 (weighted), suggesting moderate to substantial agreement.

Linear association measures demonstrated a strong correlation between machine and manual QTc-B (Pearson’s r = 0.789, Spearman’s ρ = 0.755, both p < 0.001), indicating that despite minor variations, machine and manual calculations showed a consistent relationship.

## Correlation Between Manual QTc-Bazett and QTc-Fridericia

To compare manual QTc-B and QTc-Fridericia (QTc-F) values, we performed the same statistical analyses. The ICC between QTc-B and QTc-F was 0.744 (95% CI: -0.044–0.938, p = 0.0623), reflecting good agreement but with a wide confidence interval, likely due to the small sample size of patients with bradycardia (n = 26). Cohen’s Kappa for categorical agreement was 0.00, indicating poor agreement, though this may also be an artifact of the small sample size.

Pearson’s and Spearman’s correlations both demonstrated a strong positive association between QTc-B and QTc-F (Pearson’s r = 0.964, Spearman’s ρ = 0.979, both p < 0.001), suggesting that although QTc-F may provide slightly different corrected QT interval estimates, it remains highly correlated with QTc-B.
